# Concentration–Response Function for Ozone and Daily Mortality: Results from Five Urban and Five Rural U.K. Populations

**DOI:** 10.1289/ehp.1104108

**Published:** 2012-07-19

**Authors:** Richard W. Atkinson, Dahai Yu, Ben G. Armstrong, Sam Pattenden, Paul Wilkinson, Ruth M. Doherty, Mathew R. Heal, H. Ross Anderson

**Affiliations:** 1 Division of Population Health Sciences and Education and MRC-HPA Centre for Environment and Health, St George’s, University of London, London, United Kingdom; 2 Public and Environmental Health Research Unit, London School of Hygiene and Tropical Medicine, London, United Kingdom; 3 School of GeoSciences, and; 4 School of Chemistry, University of Edinburgh, Edinburgh, United Kingdom

**Keywords:** concentration–response function, daily mortality, ozone, U.K. population

## Abstract

Background: Short-term exposure to ozone has been associated with increased daily mortality. The shape of the concentration–response relationship—and, in particular, if there is a threshold—is critical for estimating public health impacts.

Objective: We investigated the concentration–response relationship between daily ozone and mortality in five urban and five rural areas in the United Kingdom from 1993 to 2006.

Methods: We used Poisson regression, controlling for seasonality, temperature, and influenza, to investigate associations between daily maximum 8-hr ozone and daily all-cause mortality, assuming linear, linear-threshold, and spline models for all-year and season-specific periods. We examined sensitivity to adjustment for particles (urban areas only) and alternative temperature metrics.

Results: In all-year analyses, we found clear evidence for a threshold in the concentration–response relationship between ozone and all-cause mortality in London at 65 µg/m^3^ [95% confidence interval (CI): 58, 83] but little evidence of a threshold in other urban or rural areas. Combined linear effect estimates for all-cause mortality were comparable for urban and rural areas: 0.48% (95% CI: 0.35, 0.60) and 0.58% (95% CI: 0.36, 0.81) per 10-µg/m^3^ increase in ozone concentrations, respectively. Seasonal analyses suggested thresholds in both urban and rural areas for effects of ozone during summer months.

Conclusions: Our results suggest that health impacts should be estimated across the whole ambient range of ozone using both threshold and nonthreshold models, and models stratified by season. Evidence of a threshold effect in London but not in other study areas requires further investigation. The public health impacts of exposure to ozone in rural areas should not be overlooked.

Short-term exposure to ozone has been associated with a range of adverse health effects in experimental and epidemiological studies [World Health Organizaiton (WHO) 2006; Royal Society 2008]. The epidemiological evidence from individual-level panel studies has shown associations with changes in lung function in healthy subjects and symptom exacerbation and increased medication use in asthmatic subjects. By far, the most frequently reported epidemiological evidence is based on ecological time-series studies in which daily health events such as death counts are associated with ambient ozone concentrations on the same or previous days (Anderson et al. 2004; Bell et al. 2005; Ito et al. 2005; Levy et al. 2005; WHO 2006). Many of these studies are based on single cities, but multicity studies that used standardized methods have reported similar results in various continents (Bell et al. 2004; Gryparis et al. 2004; Wong et al. 2008). Current evidence suggests that short-term exposure to ozone is associated with small but significant increases in daily mortality and that this association is not an artifact of confounding by particulate matter air pollution.

The question of whether or not there is a threshold for ozone effects is critical for estimating public health impacts. Because high ozone days are relatively few, impact assessments based on days when ozone is above a threshold value yield much smaller impacts than assessments based on all days, whatever the ozone concentration (Royal Society 2008; Stedman et al. 1997). The United Nations Economic Commission for Europe (UNECE) Convention on Long-Range Trans-boundary Air Pollution recommended a threshold of 70 µg/m^3^ (35 ppb) as an 8-hr mean for the purpose of integrated assessment of health impacts by the European Union (UNECE 2003). This recommendation did not imply that effects might not occur below this level, and subsequent reviews have also concluded that time-series studies show concentration–response functions that do not exhibit a threshold (WHO 2006) or do so at daily average levels < 40 µg/m^3^ (Bell et al. 2006). Nevertheless, many studies are based primarily on ozone exposures during the warm season, when personal exposures are more highly correlated with outdoor concentrations. In addition, ozone concentrations may not be measured during cool periods, or they may be too low to estimate effects with reasonable precision.

Relatively few investigations have set out to address systematically the evidence for mortality thresholds or determine whether associations are modified by time of year (season) or confounded by coexposure to other air pollutants. Furthermore, there are few reports from rural areas. Here we report an analysis of the acute effects of short-term exposure to ozone on mortality from all causes in the five largest urban conurbations and five rural areas in England and Wales for the period 1993–2006. We focused our investigation on the shape of the concentration–response relationship between ozone and mortality, and on evidence for a threshold effect in all-year and seasonal analyses (spring, summer, fall, and winter). We also investigated whether associations in urban areas were confounded by exposure to particulate matter (PM) with an aerodynamic diameter < 10 μm (PM_10_).

## Methods

*Study populations.* Urban areas were represented by the five largest urban areas by population in England and Wales (Liverpool, London, Manchester, Tyneside, and West Midlands). To select the five rural study areas, we first assessed the availability of daily ozone data from monitors in England and Wales classified as “rural” in the Automatic Urban and Rural Network (AURN) of monitoring stations. After analyzing daily correlations between ozone concentrations at neighboring monitors, we selected the study areas to be circles with radii of 60 km centered on each monitoring station. Census districts within these circles that were classified as urban according to the U.K. Office of National Statistics (populations ≥ 10,000) (Bibby and Shepherd 2005) were excluded so that the rural populations included only small towns and villages with < 10,000 inhabitants. From all such circles, we selected five rural areas centered on the monitoring stations at Aston Hill, Harwell, High Muffles, Ladybower, and Yarner Wood that optimized the availability of daily ozone data during the study period, minimized overlap between areas, and were spread throughout England and Wales.

*Data.* Individual death records were obtained from the Office for National Statistics (Newport, Wales) and matched by postcode to the five urban and five rural populations selected for our study. Daily counts of deaths from all causes were classified according to the *International Classification of Diseases, 9th* and *10th Revisions* [ICD-9 codes > 800 for all deaths, excluding those from external causes (WHO 1975); ICD-10 codes with prefixes “E” or “Y”) (WHO 2007)] and were compiled for the period 1993–2006.

For each urban area, ozone data from urban background air quality monitoring stations were obtained from the AURN (Department for Environment Food and Rural Affairs, London, UK) and were used to construct daily maximum 8-hr running mean ozone concentrations for 1993–2006. The numbers of ozone monitoring stations that provided data in each conurbation were 1 station in Liverpool, 11 in London, 2 in Manchester, 1 in Tyneside, and 4 in West Midlands. Correlations between daily 8-hr average ozone concentrations from monitors within each city were high (*r* > 0.95). For conurbations with two or more pollution monitoring stations with at least 75% nonmissing values over the study period, daily 8-hr average ozone concentrations were calculated using the AIRGENE (Air Pollution and Inflammatory Response in Myocardial Infarction Survivors: Gene-Environment Interaction in a High Risk Group) algorithm to avoid spurious fluctuations on days on which a station did not report values (Rückerl et al. 2007). These daily, within-city averages were used as the population average exposure. PM_10_ measurements were available for urban areas only. The daily 24-hr average of PM_10_ concentrations were calculated using the same procedure for ozone described above.

Meteorological data (daily temperature and humidity) for each area were obtained from the British Atmospheric Data Centre (BADC; Harwell Oxford, Didcot, UK) for the same period and were processed using a similar procedure as described for ozone data. Where daily temperature data were unavailable, data were imputed from regional BADC series (Pattenden et al. 2010). We obtained daily counts of laboratory-confirmed cases of influenza A and respiratory syncytial virus for each study area (as a measure of the level of circulating viral infections) from the U.K. Communicable Diseases Surveillance Centre at the Health Protection Agency (Colindale, London, UK). From these daily data we calculated the 7-day moving average for each measure.

*Statistical methods.* A two-stage approach was used. First, we carried out individual area-specific analyses (five urban and five rural) that were adjusted for temporal confounders. In the second stage, the results from the five urban and the five rural populations were each pooled using meta-analysis techniques.

The relationship between daily mortality and ozone concentration was estimated using a Poisson regression model of the daily numbers of deaths conditioned on the total number of deaths in the same month. This stratification is equivalent to a time-stratified case-series analysis (Farrington and Whitaker 2006). To allow for any residual seasonality, we incorporated terms to describe an annual sinusoidal pattern in the numbers of deaths. We controlled simultaneously for the effects of mean temperature averaged on the day of and on the day before death (mean lag 0–1) and averaged across days 2–6 before death (mean lag 2–6). Season and mean temperature were modeled using natural cubic splines with six knots. Dummy variables for days during the heat wave in London during 2003 were included in the London models. In addition, models included linear terms for moving 7-day cumulative counts of influenza cases, and indicator variables for the day of the week.

We assessed the shape of the ozone mortality concentration–response relationship using three alternative model specifications: *a*) linear, *b*) linear-threshold with the threshold chosen to maximize the likelihood function, and *c*) natural cubic splines constructed using five equally spaced knots determined from the combined concentrations of ozone in the five urban areas and in the five rural areas. The mean 8-hr ozone concentration for lag 0–1 was selected *a priori* as the relevant exposure metric following previous studies (Katsouyanni et al. 2009). Model fit was compared using Akaike information criteria (AIC). Season-specific analyses were conducted based on four discrete time periods: spring (April–June), summer (July–September), fall (October–December), and winter (January–March). All analyses were conducted using Stata/SE 10 (StataCorp LP, College Station, TX, USA).

Spline curves from individual locations were combined to derive aggregated concentration–response curves for urban and rural areas using the method described by Samoli et al. (2003). In brief, knot coefficients for the spline terms for ozone were jointly meta-analyzed using the “mvmeta” procedure in Stata (White 2009). The summary ozone spline curve was then constructed at 1-μg/m^3^ increments of ozone with all covariates set at zero. For models including linear terms of ozone, the pooled ozone coefficients were estimated by inverse variance methods (Harris et al. 2008).

Various sensitivity analyses were conducted. First, we assessed potential confounding by particulate matter air pollution by rerunning the seasonal analyses incorporating mean lag 0–1 PM_10_ concentrations. These two-pollutant models were restricted to the urban conurbations where daily measures of PM_10_ were available. Second, we assessed the impact of adjusting for daily maximum temperature rather than daily mean temperature. Finally, to assess the evidence for an ozone association independent of temperature, we stratified days during the summer months by 2°C mean temperature “bins” and plotted the concentration–response relationship between risk of death and ozone separately for each bin in London and West Midlands, the two largest urban study areas.

## Results

Median numbers of daily deaths from all disease-related causes ranged from 155/day in London to 18/day in Tyneside, and from 11 to 28 deaths/day in the rural populations (Table 1).

**Table 1:** Descriptive statistics for daily mortality, concentrations of ozone and PM_10_, and average temperature for five urban and five rural areas, 1993–2006.

Study area	Deathsa (n/day)	Temperatureb (°C)	Ozonec (μg/m3)											PM10d (μg/m3)		
			All year	Spring	Summer	Fall	Winter									
	Median	5th, 50th, 95th	N	5th, 50th, 95th	5th, 50th, 95the	5th, 50th, 95th	5th, 50th, 95th	5th, 50th, 95th	N	Median
Urban
Liverpool	19	2.2, 10.4, 17.9	3,227	10, 54, 85	37, 66, 91	25, 53, 87	6, 38, 71	7, 53, 79	3,186	25.0
London	155	2.5, 11.2, 20.2	5,092	11, 49, 90	37, 66, 102	26, 53, 112	5, 30, 62	9, 43, 70	5,098	24.2
Manchester	42	2.0, 10.3, 18.5	3,993	12, 48, 81	41, 62, 94	27, 47, 96	6, 35, 60	10, 47, 70	4,006	22.3
Tyneside	18	2.3, 10.0, 18.0	4,077	16, 54, 85	41, 66, 94	27, 49, 88	7, 43, 71	12, 58, 82	4,802	20.0
West Midlands	63	1.4, 10.0, 18.7	5,100	13, 54, 90	41, 68, 103	30, 55, 114	7, 39, 66	11, 52, 77	5,041	21.5
Rural
Aston Hill	13	1.3, 9.4, 17.3	4,723	43, 74, 109	64, 86, 122	52, 69, 136	31, 66, 81	39, 75, 91	—	—
Harwell	23	1.5, 10.1, 18.7	4,696	25, 68, 114	58, 83, 127	45, 70, 139	10, 55, 76	19, 66, 85	—	—
High Muffles	11	1.2, 9.4, 17.9	4,775	39, 69, 108	60, 85, 119	48, 67, 123	25, 59, 75	37, 71, 92	—	—
Ladybower	28	1.3, 9.4, 17.9	4,553	33, 65, 99	58, 79, 113	38, 61, 125	22, 55, 74	27, 67, 86	—	—
Yarner Wood	14	2.3, 9.9, 17.2	4,737	40, 72, 110	62, 87, 127	47, 68, 129	29, 64, 79	36, 74, 93	—	—
Abbreviations: —, data not available; N, number of days with available data. 5th ,50th, and 95th represent percentiles of the distribution. aMedian number of deaths from all disease-related causes. bMean daily temperature. cDaily maximum 8-hr mean. dDaily mean PM10.																				

Daily maximum 8-hr ozone concentrations (abbreviated to “ozone concentrations” in the subsequent text) were higher in rural areas than in urban areas (Table 1). Median, all-year ozone concentrations were between 48 and 54 μg/m^3^ in urban areas and between 65 and 74 μg/m^3^ in rural areas. The highest daily urban and rural ozone concentrations were 199 μg/m^3^ in London and 222 μg/m^3^ in Yarner Wood in the South West region of England (data not shown). Median ozone concentrations were highest in spring and lowest in the fall in both rural and urban areas (Table 1). Median summer ozone concentrations were generally comparable to median winter concentrations. Daily average temperatures were higher in the urban versus rural areas (Table 1), reaching > 28°C in London during the heat wave in 2003, compared with a maximum daily average temperature of 25°C in Harwell in central England (data not shown). Median daily average concentrations of PM_10_ ranged from 20 μg/m^3^ in Tyneside to 25 μg/m^3^ in Liverpool (Table 1). Pearson correlations between ozone and PM_10_ were negative during the fall and winter months (–0.62 to –0.52 and –0.59 to –0.40, respectively) and generally positive during the spring and summer months (–0.04 to 0.28 and 0.14 to 0.59, respectively).

Results for individual locations for all-year analyses assuming linear, linear-threshold, and spline models are given in Table 2 and individual concentration–response curves derived from the spline models are illustrated in Supplemental Material, Figure S1 (http://dx.doi.org/10.1289/ehp.1104108). In all urban areas, except London, there was little to distinguish linear models from either the linear-threshold or spline models: goodness-of-fit statistics were comparable (within-area changes in AIC were small), and optimum threshold values were near the lower ends of the ozone concentration ranges, resulting in above-threshold slope estimates that were comparable to estimates from linear models. In London, however, there was evidence for a threshold estimated at an ozone concentration of 65 μg/m^3^ [95% confidence interval (CI): 58, 83]. Consequently, combined urban concentrations–response curves were heterogeneous (*p* = 0.01), and the summary curve for the full year analysis is not presented. Assuming linearity, a 10-µg/m^3^ increase in 2-day mean daily ozone (lag 0–1) was associated with a 0.48% (95% CI: 0.35, 0.60) increase in daily mortality based on the combined random effects summary estimate for the five urban areas. We found no evidence of heterogeneity (*p* = 0.99) between the individual concentration–response curves for the five rural areas [see Supplemental Material, Figure S2 (http://dx.doi.org/10.1289/ehp.1104108)] and little evidence of a nonlinear association (Table 2) with the exception of Aston Hill, where an effect threshold at 88 μg/m^3^ (95% CI: 6, 134) was estimated. The combined linear effect estimate for the rural areas indicated a 0.58% (95% CI: 0.36, 0.81) increase in daily all-cause mortality per 10-µg/m^3^ increase in 2-day average ozone concentrations.

**Table 2:** Results from analyses that assumed linear, linear-threshold, and spline models for all-cause mortality.

Area	Linear model			Threshold model							Spline model		
				Ozone range (μg/m^3^)	Ozone threshold [μg/m^3^ (95% CI)]	Percent (95% CI)^c^	ΔAIC^d^	Linearity p-value^e^	ΔAIC^d^
	Percent (95% CI)^a^	AI^b^							
Urban
Liverpool	0.72 (0.19, 1.26)	17,553	3.0–142.0	6 (3, 122)	0.73 (0.19, 1.27)	5.9	0.08	–0.9
London	0.38 (0.22, 0.55)	39,427	1.7–178.2	65 (58, 83)	1.33 (0.80, 1.86)	–23.8	0.00	–20.6
Manchester	0.68 (0.28, 1.07)	25,055	1.6–148	6 (1, 23)	0.68 (0.29, 1.07)	6.0	0.06	–1.5
Tyneside	0.50 (–0.02, 1.02)	21,749	2.0–154.5	2 (2, 155)	0.50 (–0.02, 1.02)	6.0	0.51	3.7
West Midlands	0.55 (0.30, 0.80)	34,444	2.4–173.2	2 (2, 27)	0.55 (0.30, 0.80)	6.0	0.11	–0.1
Summary estimate	0.48 (0.35, 0.60)
Rural
Aston Hill	0.42 (–0.19, 1.03)	23,626	6–209.5	88 (6, 134)	1.31 (0.22, 2.41)	2.3	0.29	2.2
Harwell	0.54 (0.13, 0.94)	26,083	2–193	12 (2, 119)	0.55 (0.14, 0.96)	5.8	0.17	0.9
High Muffles	0.29 (–0.36, 0.94)	23,384	2.5–185.5	181 (2, 186)	NAf	5.5	0.09	–0.5
Ladybower	0.86 (0.41, 1.31)	25,971	3–187.5	3 (3, 66)	0.86 (0.41, 1.31)	6.0	0.85	5.2
Yarner Wood	0.59 (0.04, 1.15)	24,074	2.5–220	2 (2, 219)	0.59 (0.04, 1.15)	6.0	0.06	–1.4
Summary estimate	0.58 (0.36, 0.81)
aPercent increase in daily all-cause mortality per 10-µg/m3 increase in maximum 8-hr ozone concentrations on the current day and previous day. bAIC for models including linear term for ozone. cPercent increase in daily all-cause mortality per 10-µg/m3 increase above threshold in maximum 8-hr ozone concentrations on the current day and previous day. dChange in AIC from linear model (negative indicates better fit than linear). ep-Value test for departure from linearity. fInsufficient data to estimate coefficient above threshold.																

**Figure 1:**
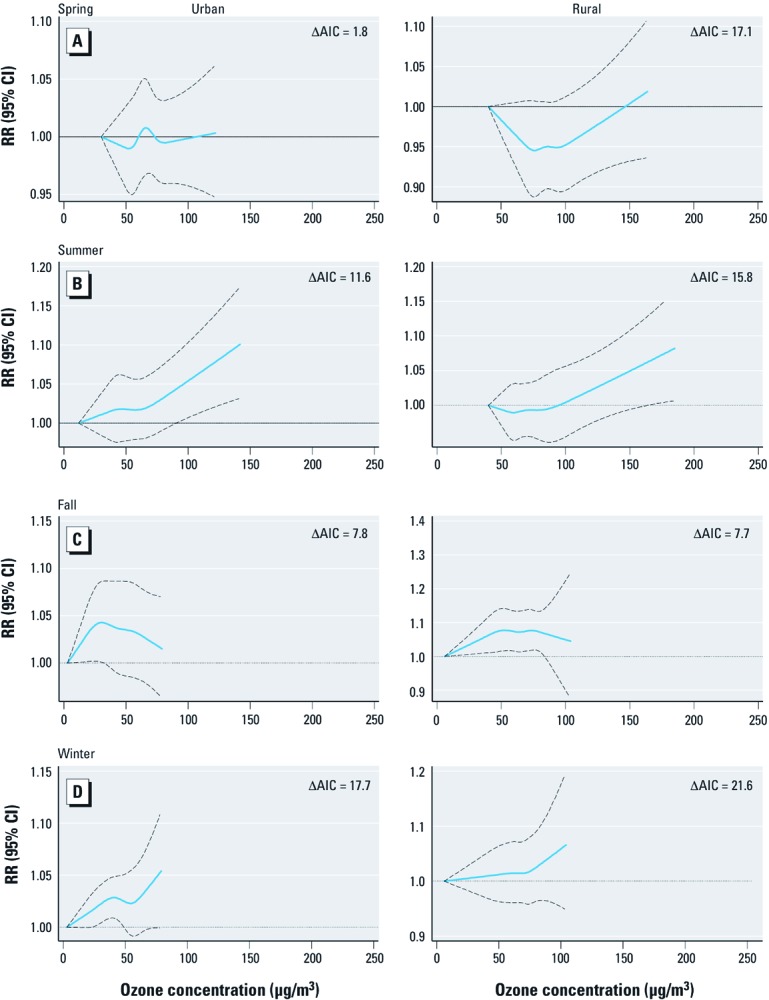
Season-specific combined concentration–response curves for ozone and mortality from all-causes for the five urban (left) and five rural (right) areas: (*A*) spring, (*B*) summer, (*C*) fall, and (*D*) winter. ΔAIC, change in AIC from linear to spline model. Values shown are relative risk of death and 95% CIs associated with ozone concentration. Summary curves are evaluated up to the minimum (across areas) maximum daily ozone concentrations. Spring, April–June; Summer, July–September; Fall, October–December; Winter, January–March.

Summary concentration–response curves for the five urban and five rural areas during spring, summer, fall, and winter are illustrated in Figure 1. Summary estimates of the associations assuming linear and linear-threshold models are given in Table 3. Model estimates suggest a threshold in the concentration–response relationship for mortality in both urban (64 μg/m^3^; 95% CI: 56, 73) and rural (79 μg/m^3^; 95% CI: 56, 101) areas during the summer months, although model fit was not significantly better for spline versus linear no-threshold models (Figure 1). Combined linear effect estimates for daily mortality during the summer, which assumed no threshold, were 0.65% (95% CI: 0.39, 0.91) and 0.46% (95% CI: –0.01, 0.92) per 10-µg/m^3^ increase in ozone concentrations in urban and rural areas, respectively, whereas corresponding above-threshold effect estimates were 1.10% (95% CI: 0.71, 1.49) and 0.82% (95% CI: 0.22, 1.43) (Table 3).

**Table 3:** Estimates of summary linear ozone effect for all-cause mortality, assuming linear and threshold models and sensitivity analyses, using alternative temperature metrics (mean or maximum temperature) and adjusting for PM_10_.

Area/model	Model	Spring	Summer	Fall	Winter
Urban
Mean temperature	O3 linear [% (95% CI)]a	0.13 (–0.14, 0.39)	0.65 (0.39, 0.91)	0.42 (–0.29, 1.12)	0.44 (0.13, 0.76)
Threshold [μg/m3 (95% CI)]	110 (83, 137)	64 (56, 73)	11 (0, 21)	33 (3, 64)
O3 linear > threshold [% (95% CI)]a	0.07 (–1.74, 1.89)	1.10 (0.71, 1.49)	0.39 (–0.33, 1.11)	0.40 (0.05, 0.75)
Maximum temperature	O3 linear [% (95% CI)]a	–0.06 (–0.35, 0.22)	0.21 (–0.10, 0.52)	0.42 (–0.29, 1.13)	0.45 (0.14, 0.75)
Threshold [μg/m3 (95% CI)]	NAb	97 (81, 112)	NAb	NAb
O3 linear > threshold [% (95% CI)]a	NAb	0.66 (–0.27, 1.58)	NAb	NAb
Mean temperature + PM10c	O3 linear [% (95% CI)]a	0.15 (–0.12, 0.42)	0.62 (0.35, 0.90)	0.05 (–0.54, 0.65)	0.13 (–0.21, 0.47)
Rural
Mean temperature	O3 linear [% (95% CI)]a	0.25 (–0.23, 0.72)	0.46 (–0.01, 0.92)	0.62 (0.08, 1.16)	0.39 (–0.13, 0.91)
Threshold [μg/m3 (95% CI)]	130, (97, 162)	79 (56, 101)	42 (6, 77)	76 (46, 106)
O3 linear > threshold [% (95% CI)]a	0.72 (–1.98, 3.42)	0.82 (0.22, 1.43)	0.49 (–0.36, 1.31)	1.02 (–0.82, 2.87)
Maximum temperature	O3 linear [% (95% CI)]a	0.11 (–0.44, 0.65)	0.18 (–0.32, 0.69)	0.58 (0.07, 1.10)	0.42 (–0.10, 0.94)
Threshold [μg/m3 (95% CI)]	NAb	136 (100, 172)	NAb	NAd
O3 linear > threshold [% (95% CI)]a	NAb	0.46 (–1.21, 2.14)	NAb	NAd
aRandom effect summary estimate percent increase in daily mortality per 10-μg/m3 increase in 8-hr maximum ozone concentrations on the current day and on the previous day. bNot appropriate because linear concentration–response relationship assumed. cModel includes natural cubic spline for PM10 average lag 0–1.										

The strongest evidence for a threshold in the ozone–mortality relationship among individual study areas during the summer was found in the large urban conurbations (London, Manchester, and the West Midlands) [see Supplemental Material Table S1 and Figure S2 (http://dx.doi.org/10.1289/ehp.1104108)]. In London, ozone concentrations above a threshold of 64 µg/m^3^ (95% CI: 56, 74) were associated with a 1.35% (95% CI: 0.78, 1.88) increase in daily mortality per a 10-µg/m^3^ increase in maximum 8-hr ozone concentrations (lag 0–1).

We found little evidence for a threshold in the other seasons (Figure 1 and Table 3). There was little to distinguish linear models from either the linear-threshold or spline models: Goodness-of-fit statistics for the spline models increased slightly from the linear models, and the above-threshold slope estimates were comparable to estimates from linear models. Combined linear effect estimates for urban areas during the spring, fall, and winter seasons were 0.13% (95% CI: –0.14, 0.39), 0.42% (95% CI: –0.29, 1.12), and 0.44% (95% CI: 0.13, 0.76) per 10 µg/m^3^, respectively.

In both urban and rural areas, adjusting for maximum temperature instead of mean temperature attenuated the effect estimate for the summer season but made little overall difference in the spring, fall, and winter months (Table 3). In urban areas, adjusting for PM_10_ attenuated the estimates of the summary ozone linear effect in the fall and winter season-specific analyses, but not in the spring or summer periods.

**Figure 2:**
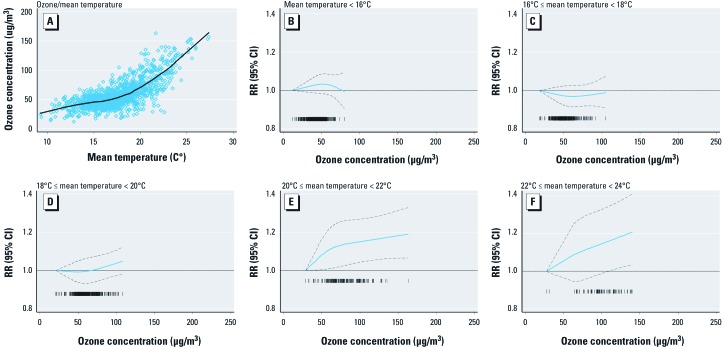
Sensitivity analyses of the ozone–mortality relationship for London during the summer months. (*A*) Scatter plot of ozone concentrations versus mean temperature for study days, 1993–2006. (*B–F*) Relative risk of death and 95% CIs associated with ozone concentration for mean temperatures (*B*) < 16°C, (*C*) ≥ 16°C but < 18°C, (*D*) ≥ 18°C but < 20°C, (*E*) ≥ 20°C but < 22°C, (*F*) ≥ 22°C but < 24°C.

Results of further sensitivity analyses of data for the summer period for London, the largest and most informative city in our analysis, are illustrated in Figure 2. Ozone concentrations were closely correlated with mean daily temperature (Figure 2A), with the rate of increase in ozone concentrations per degree Celsius increase in daily mean temperature changing substantially at approximately 18°C. Analyses of days stratified by 2°C bins of mean temperature (Figure 2B–F) suggested effect modification of the ozone–mortality relationship by temperature. Specifically, there appeared to be no relationship between ozone and mortality on days with mean temperatures below 20°C (Figure 2B–D), but there was clear evidence of a relationship on days with mean temperatures above 20°C (Figure 2E–F). Corresponding estimates for the West Midlands area, the next largest urban conurbation in the United Kingdom, were similar (data not shown). Analyses of London data for the spring months also suggested effect modification of the ozone–mortality relationship by temperature (data not shown).

## Discussion

In this study we examined associations between daily measures of ozone and daily mortality in five urban and five rural areas of England and Wales. We focused our investigation upon the shape of the concentration–response relationship, assessing evidence for nonlinearity and the existence of a threshold effect. In our all-year analysis we found evidence to reject the assumption of a linear association only in London. Season-specific analyses however provided evidence for nonlinearity during summer months in both urban and rural areas. We also observed linear associations with mortality during fall and winter that were attenuated on adjustment for PM_10_. We found little evidence for a relationship between ozone and mortality during spring months.

Few studies have set out to investigate specifically the shape of the concentration–response relationship between ozone levels and daily mortality. Bell et al. (2006) studied the relationship with mortality in 98 U.S. communities and reported a threshold at 20 μg/m^3^ (10 ppb) for 24-hr average ozone, with associations that were approximately linear above this concentration. Kim et al. (2004) reported a threshold around 16–24 μg/m^3^ (8–12 ppb) for daily 24-hr ozone (scaled from 1-hr maximum concentration) in Seoul, Korea. An alternative approach used in some studies has been to repeat analyses using days with high values excluded, an approach designed to identify the existence of a threshold rather than nonlinearity in general. Hoek et al. (1997) reported that relative risk estimates for mortality associated with daily changes in ozone were robust to exclusion of days with 24-hr averages ≥ 40 μg/m^3^ in a study of Rotterdam, the Netherlands. They concluded that should a threshold exist, it may be at low concentrations. Bell et al. (2006) also tried this approach and drew the same conclusion. The most recent analyses of data from the Air Pollution and Health: A European and North American Approach (APHENA) study, a multicity study from North America, Canada, and Europe did not find evidence of a threshold-exposure level for the association between ozone and mortality (Katsouyanni et al. 2009). The authors attributed this to inadequate statistical power to estimate thresholds with their time-series data. Overall, the evidence for a nonlinear concentration–response relationship between daily ozone and deaths, and in particular the existence of a threshold for response, is rather weak. If a threshold does exist then it would seem to be at low ozone concentrations. Our all-year results were broadly comparable with the existing literature, with the notable exception of London, for which there was evidence of a relatively high threshold at 65 μg/m^3^.

We are uncertain why a threshold would be present in London but not in other areas. London’s large population may have provided the statistical power needed to identify a threshold. Alternatively, the heat island effect is greater in London than elsewhere (Hajat et al. 2007) and, given the mild U.K. climate, may lead to the population spending less time indoors than in other cities. This would affect a population’s exposure to air pollution leading to differential exposure misclassification (Brauer et al. 2002). Also, the role of temperature in modifying the ozone–mortality relationship has been shown to differ by geographical region (Ren et al. 2008). Finally, the air pollution mixture on high ozone days in London may be different from the air pollution mixture on low ozone days; if so, high ozone concentrations may be acting as a marker for other pollutants (or a mixture of pollutants) that might be responsible for the observed health effects. We could not investigate this hypothesis further because of the limited data for other pollutants (only PM_10_ was available in urban areas). However, an analysis of additional pollutant data available for London for a shorter period of time (2000–2005) indicated that high ozone days are also days with high secondary particles (particularly nitrates) and low nitrogen dioxide, carbon monoxide, particle number concentrations and chlorides (data not shown). Also, London is unique in the United Kingdom in that its geographical proximity to mainland Europe leads to a greater impact from “continental” pollution than in other U.K. cities, with the possible exception of West Midlands (SNIFFER 2011).

Our season-stratified analyses revealed evidence for different patterns of concentration–response function for each season: *a*) evidence for a threshold in both urban and rural areas during summer months, *b*) little evidence of associations during spring months (characterized by the highest ozone concentrations), and *c*) evidence of linear associations (without thresholds) between ozone and mortality during cooler periods of the year (fall and winter). Seasonal variation in associations between ozone and mortality has been reported previously (Bell et al. 2005; Gryparis et al. 2004; Ito et al. 2005; Levy et al. 2005), but threshold effects were not evaluated in these studies. Brauer et al. (2002) suggested that seasonal variation in thresholds might be explained by differential exposure misclassification that arises from seasonal variations in the ratio of indoor:outdoor activity, the ratios of indoor:outdoor pollution, or from meteorological and atmospheric chemistry conditions. The occurrence of a spring maximum in the annual ozone cycle is a well-known observational phenomenon throughout the troposphere, especially on the western edge of mainland Europe (Monks 2000). The source of ozone during the winter months (extending into spring) in the United Kingdom is predominantly Northern Hemisphere long-range transport (with longer lifetime of ozone) supplemented with local photochemical production (Derwent 2008; Derwent et al. 2004). In the summer, meteorological conditions are conducive to the “local” production of ozone and may also be associated with secondary PM pollution (Royal Society 2008).

We explored the possible role of seasonal differences in the relationship between particulate matter (PM_10_) and ozone concentrations in urban areas, and observed no attenuation of the ozone effect estimates in summer months—a finding consistent with previous reports by Katsouyanni et al. (2009). The attenuation of the fall and winter ozone associations following adjustment for PM_10_ was expected given the negative correlation between ozone and PM in the winter. Adjusting for PM_10_ in models for spring had little impact on the size and precision of the effect estimates for ozone and mortality. The lack of data on particles, particularly secondary particles that are closely correlated with ozone, remains a significant shortcoming of our study.

Our sensitivity analyses using daily maximum temperature (vs. mean temperature) led to a decrease in the size of the associations between ozone and mortality during the spring and summer periods. This is not too surprising given the stronger correlation between ozone and maximum temperature compared with mean temperature (e.g., in London, Pearson correlation coefficient *r* = 0.78 and 0.65, respectively). The modification of ozone–mortality associations by maximum temperature has been noted previously both in the United Kingdom (Pattenden et al. 2010) and the United States (Ren et al. 2008).

Our analyses of ozone and all-cause mortality that assumed linearity yielded effect estimates comparable with other results from the literature. A systematic review and meta-analysis of the published time-series literature produced for the U.K. Department of Health (Anderson et al. 2007) reported a meta-analytic summary estimate for deaths from all causes (20 single-city estimates) of 0.22% (95% CI: 0.09, 0.35) per 10-µg/m^3^ increase in daily maximum 8-hr average ozone, although there was clear evidence of a small study bias (Sutton et al. 2000) and heterogeneity between estimates. The recent and comprehensive re-analysis of data from multiple cities in the United States, Canada, and Europe (Katsouyanni et al. 2009) reported all-year linear coefficients for all-cause mortality, expressed as a percentage change in the mean number of deaths per 10-μg/m^3^ increments in daily maximum 8-hr ozone (lags 0 and 1), of 0.32% (95% CI: 0.12, 0.52) across 54 U.S. cities, 0.97% (95% CI: 0.67, 1.3) across 12 Canadian cities, and 0.12% (95% CI: –0.02, 0.26) across 23 European cities (results quoted are from models using natural cubic splines with 12 degrees of freedom per year). Bell et al. (2004, 2005) reported results from the National Morbidity, Mortality, and Air Pollution Study (NMMAPS) database, as well as summarized published meta-analyses that were conducted by Anderson et al. (2004), Levy et al. (2001), Stieb et al. (2003), and Thurston and Ito (2001). Together with a further review and meta-analysis by Smith et al. (2009), estimates of the association between ozone and mortality range from approximately 0.3% to 0.6% per 10-μg/m^3^ increments in mean ozone. These results are comparable with the summary linear estimates obtained from our analysis of five urban and five rural areas. We report comparable associations between ozone and mortality in rural and urban areas. The existing time-series literature focuses overwhelmingly on the study of urban populations and the public health significance of exposure to outdoor air pollution in sizeable rural populations should not be overlooked.

The observational nature of the evidence presented in this study prevents one from concluding that associations between increases in daily ozone concentrations and death are causal and also suggests caution in interpreting the evidence on thresholds. Indeed, our sensitivity analyses indicate that the observed associations may be subject to some residual confounding by both PM (in urban areas) and temperature, despite extensive adjustment for the temperature in our models. Also, the complex processes involved in the production, loss, and deposition of tropospheric ozone, including effects of meteorological factors that also affect the concentrations of PM and other anthropogenic pollutants, suggest that epidemiological investigations may benefit from more sophisticated modeling of meteorological and atmospheric parameters. Thus, although the preponderance of evidence from this study points to adverse effects from low concentrations, the evidence from London, as well as other large conurbations in the United Kingdom, and the caveats above (i.e., large conurbations in the United Kingdom and the uncertainties arising from the sensitivity analyses), suggest caution is warranted when drawing conclusions. A further caveat regarding the evidence presented in this study is our focus on acute (lag 0–1) ozone exposures only, excluding potential effects at longer lags. We recognize that an individual’s exposure to ozone comprises exposure accumulated over many days. We also note the recent work of Gasparrini et al. (2010) using distributed lag, nonlinear models and see merit in pursuing this approach with our data in the future.

European health impact assessments of the short-term effects of ozone on mortality have generally adopted a threshold approach, although some have also estimated nonthreshold impacts (Anderson et al. 2007; Commission of the European Communities 2005; Royal Society 2008). The assumption of a threshold has an important influence on the estimated impact, because the lower the threshold value, the greater the proportion of days included in the calculations of attributable deaths. The assumption of a threshold may also affect predictions of future impacts because there is a trend toward a reduction in episodes of high ozone levels while background concentrations are slowly rising (Air Quality Expert Group 2009). The recommendation of a threshold 70 μg/m^3^ (35 ppb) 8-hr average by the UNECE (2003) reflected uncertainties concerning the shape of the concentration–response function below this level. Our results for London are consistent with this value, although the totality of evidence suggests that the assumption of linearity is appropriate for U.S. and Canadian health impact assessments (U.S. Environmental Protection Agency 2011). Furthermore, given the seasonal variation in the correlation between PM and ozone, impact assessment using PM-adjusted ozone coefficients would seem appropriate.

## Supplemental Material

(258 KB) PDF
