# Supplementary material for: Concentration–Response Function for Ozone and Daily Mortality: Results from Five Urban and Five Rural U.K. Populations
Source: Environ Health Perspect. 2012 Jul 19;120(10):1411–7. doi: 10.1289/ehp.1104108 (PMC3491921; doi:10.1289/ehp.1104108)
Supplement: (258 KB) PDF [file ehp.1104108.s001.pdf]

Supplemental Material

Concentration-Response Function for Ozone and Daily Mortality: Results from Five Urban and Five Rural UK Populations

Richard W Atkinson[1], Dahai Yu[1], Ben G Armstrong[2], Sam Pattenden[2], Paul Wilkinson[2], Ruth M Doherty[3], Mathew R Heal[4], H Ross Anderson[1]

1 - Division of Population Health Sciences and Education and MRC-HPA Centre for Environment and Health, St George's, University of London, London

2 - Public and Environmental Health Research Unit, London School of Hygiene and Tropical Medicine, London

3 - School of GeoSciences, University of Edinburgh, Edinburgh

4 - School of Chemistry, University of Edinburgh, Edinburgh

Contents

Supplemental Material, Figure S1 2

Supplemental Material, Table S1 4

Supplemental Material, Figure S2 5

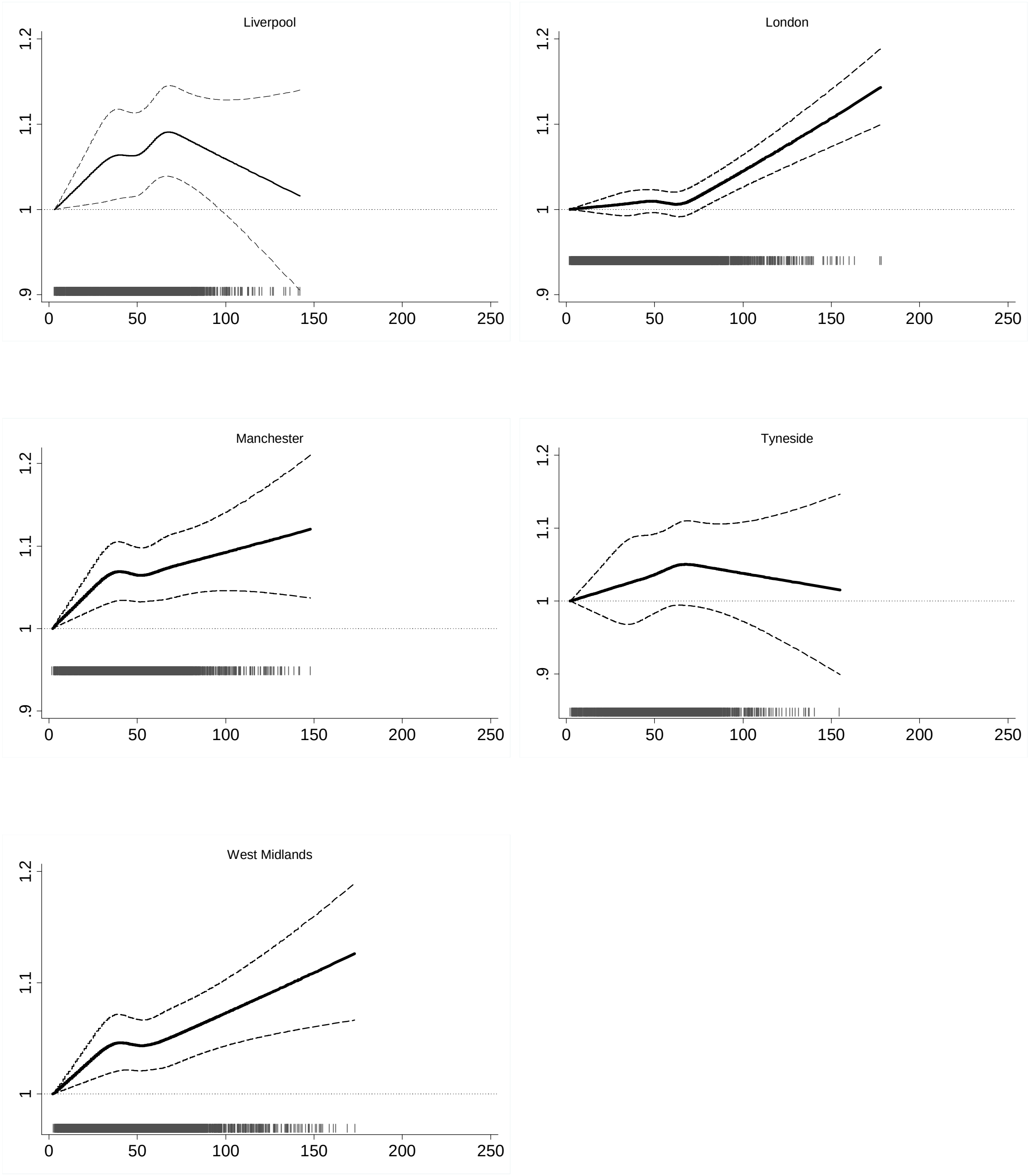

Notes:x-axis- ozone concentration in µg/m^3; y-axis relative risk of death (solid line) and 95% confidence limits (dotted line). Vertical bars indicate location of data points

Supplemental Material, Figure S1a. Concentration-response curves for ozone and all-cause mortality, all-year analysis, urban areas

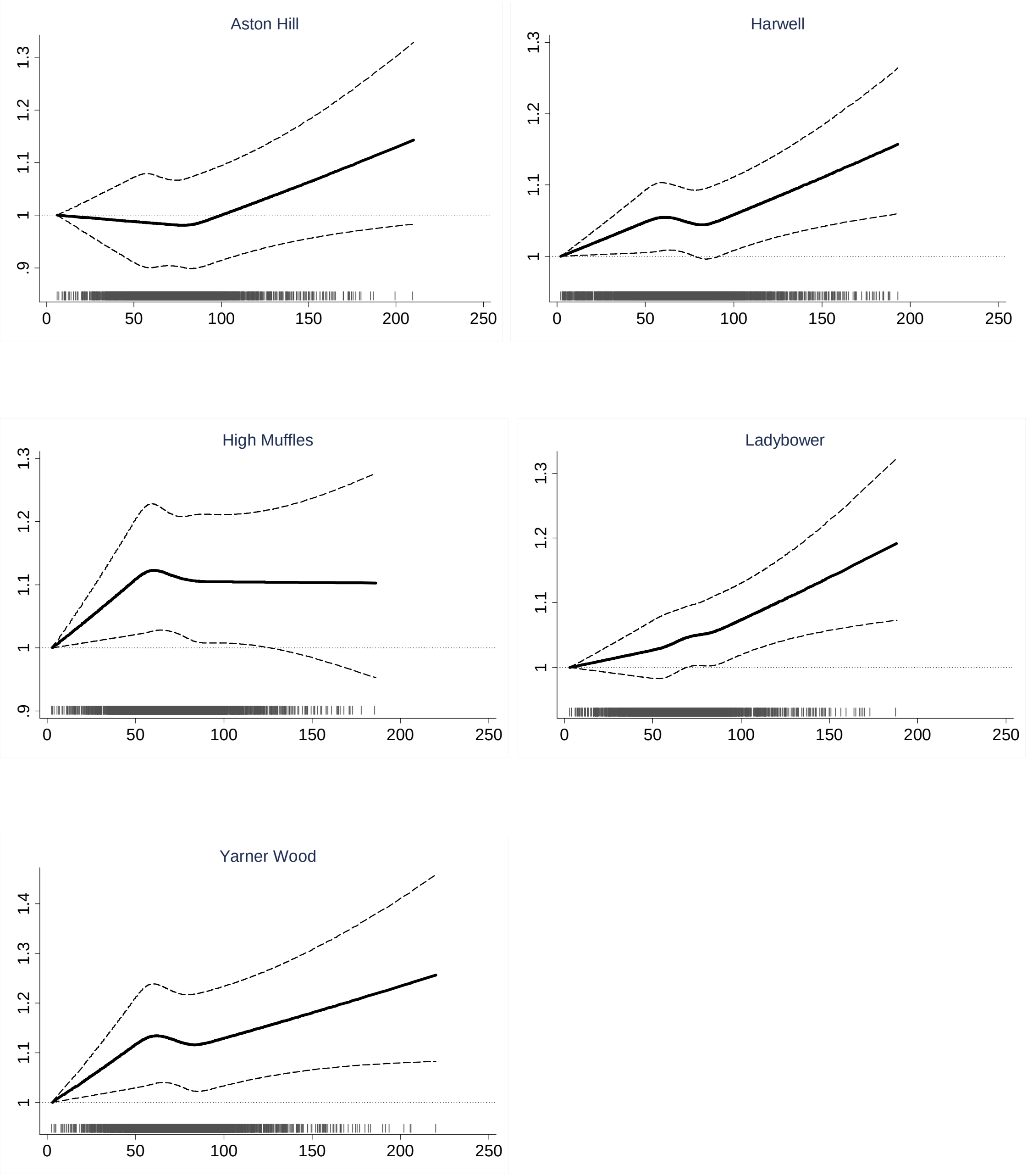

Notes: x-axis- ozone concentration in µg/m^3; y-axis relative risk of death (solid line) and 95% confidence limits (dotted line). Vertical bars indicate location of data points

Supplemental Material, Figure S1b. Concentration-response curves for ozone and all-cause mortality, all-year analysis, rural areas

Area	Linear model		Threshold Model				Spline model	
	% (95% CI)[a]	AIC	Ozone Range (µg/m³)	Threshold Estimate (95% CI) (µg/m³)	% (95% CI)[a] (above threshold)	Δ AIC[b]	P-value[c]	Δ AIC[b]
**Urban**								
**Liverpool**	0.28 (-0.90, 1.47)	4413	(10,142)	116 (10, 142)	-10.26 (-20.34, 1.10)	-3.2	0.95	5.7
**London**	0.81 (0.44, 1.17)	9610	(12.9,163.1)	64 (56, 74)	1.35 (0.82, 1.88)	-6.4	0.02	-4.4
**Manchester**	0.66 (-0.18, 1.51)	6150	(8.7,141.8)	64 (8, 100)	1.71 (0.27, 3.16)	-3.0	0.21	1.4
**Tyneside**	0.53 (-0.60, 1.67)	5232	(15.5,154.5)	38 (15, 155)	1.71 (0.50, 2.93)	-0.3	0.86	5.3
**West Midlands**	0.44 (-0.07, 0.95)	8497	(13.6,173.2)	58 (13, 165)	0.71 (0.07, 1.35)	-1.9	0.51	3.7
**Rural**								
**Aston Hill**	0.71 (-0.29, 1.72)	5520	(24.5,209.5)	86 (24, 152)	1.58 (0.04, 3.14)	-2.1	0.33	2.6
**Harwell**	0.32 (-0.42, 1.07)	6546	(25.5,193)	87 (25, 192)	1.20 (0.11, 2.29)	-3.9	0.07	-0.9
**High Muffles**	-0.37 (-1.53, 0.81)	6032	(40,185.5)	166 (40, 186)	-25.01 (-46.48, 5.07)	-2.8	0.99	5.9
**Ladybower**	1.17 (0.31, 2.03)	5875	(22,187.5)	53 (22, 78)	1.38 (0.46, 2.32)	-1.5	0.29	2.3
**Yarner Wood**	0.20 (-0.72, 1.12)	6044	(32,220)	193 (32, 220)	-12.39 (-27.47, 5.82)	-1.9	0.99	5.9

Notes: a – % per 10µg/m³increase in ozone concentration (95% confidence interval); b – Change in AIC from Linear model; c – P-value test for linearity

Supplemental Material, Table S1. Results from analyses assuming linear, linear threshold and spline models for all-cause mortality for summer months

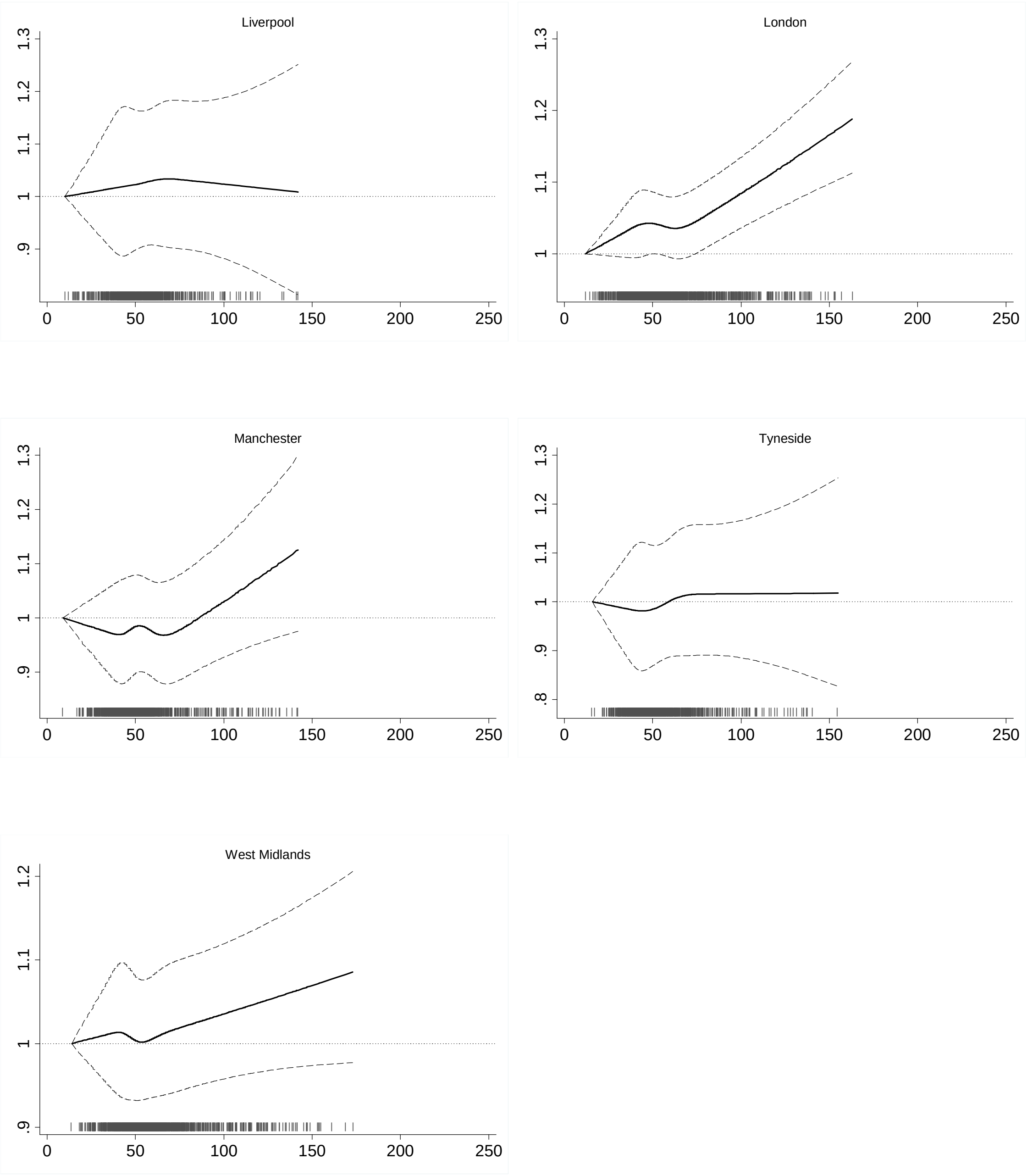

Notes: x-axis- ozone concentration in $\mu g/m^3$; y-axis relative risk of death (solid line) and 95% confidence limits (dotted line). Vertical bars indicate location of data points

Supplemental Material, Figure S2a. Concentration-response curves for ozone and all-cause mortality during summer months, urban areas

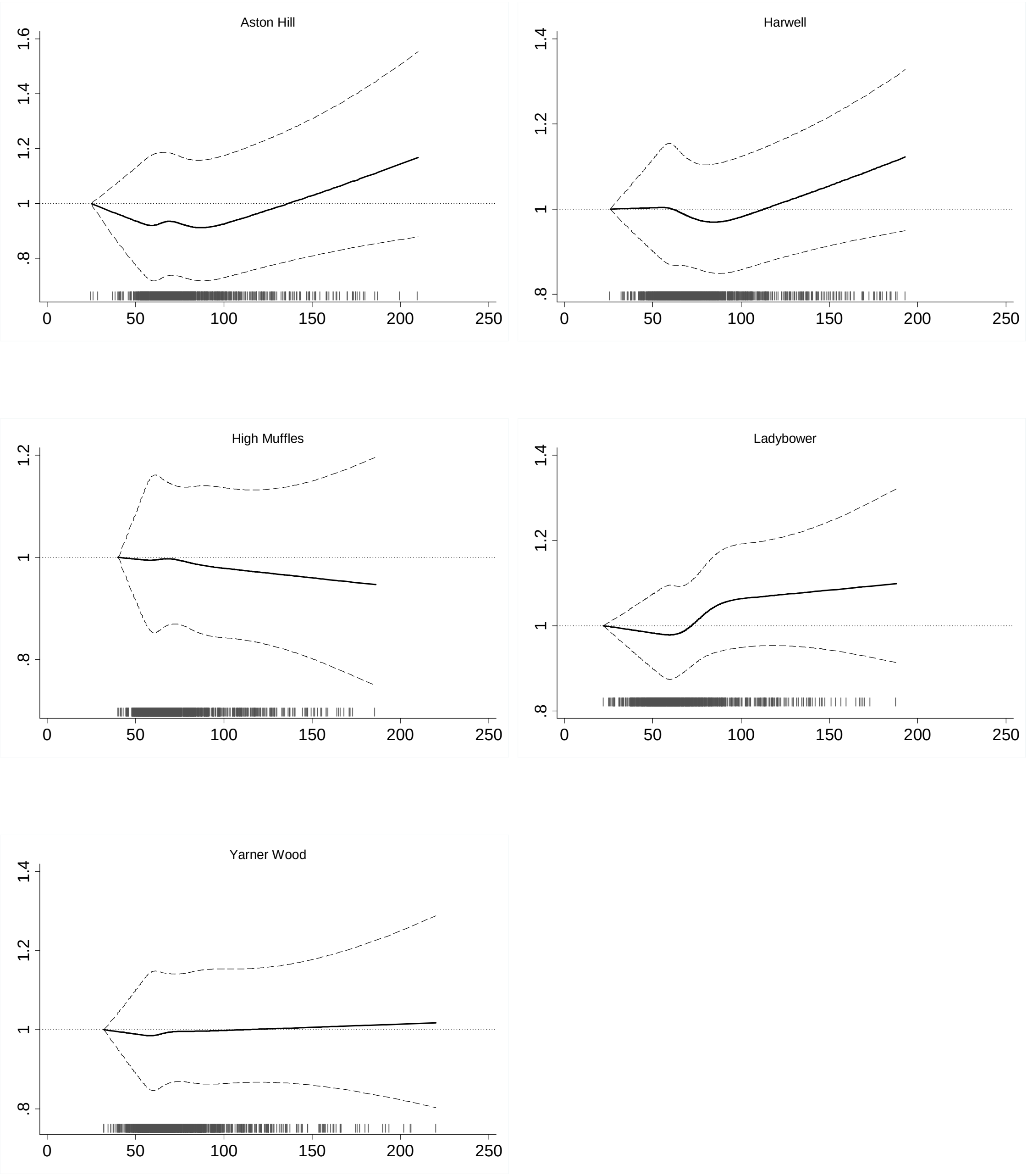

Notes: x- axis- ozone concentration in µg/m^3; y-axis relative risk of death (solid line) and 95% confidence limits (dotted line). Vertical bars indicate location of data points

Supplemental Material, Figure S2b. Concentration-response curves for ozone and all-cause mortality during summer months, rural areas
